# What can we learn from honey bees?

**DOI:** 10.7554/eLife.72380

**Published:** 2021-08-31

**Authors:** Julia A Schwartzman

**Affiliations:** Department of Civil and Environmental Engineering, Massachusetts Institute of Technology Cambridge United States

**Keywords:** honey bee, lactobacillus firm5, gut microbiota, bacterial coexistence, niche partitioning, microbiome, Other

## Abstract

The Western honey bee provides a model system for studying how closely related species of bacteria are able to coexist in a single community.

**Related research article** Brochet S, Quinn A, Mars RA, Neuschwander N, Sauer U, Engel P. 2021. Niche partitioning facilitates coexistence of closely related honey bee gut bacteria. *eLife* **10**:e68583. doi: 10.7554/eLife.68583

The gut of an animal contains a staggering amount of microbial diversity (Sankar et al., 2015). However, the species present are often members of only a few taxonomic groups and therefore tend to share many metabolic and physiological features. This observation has fascinated microbial ecologists for decades, as it has long been believed that species that are similar can only coexist if they avoid directly competing for shared resources (Gause, 1934; Hardin, 1960; Schoener, 1974; Volterra, 1926).

Recently, several theories have been put forward to explain how related species of bacteria are able to coexist in complex communities such as the gut microbiota of animals (Caetano et al., 2021; Erez et al., 2020; Goyal et al., 2018). This has led to the hypothesis that a process called resource partitioning – that is, when different species use resources in different ways to avoid competition – allows similar bacterial species to live together in a single community. However, finding an experimental system where it is possible to disentangle the confounding effects of diet, host, and microbial interactions is deceptively difficult. Now, in eLife, Philipp Engel and co-workers from the University of Lausanne and ETH Zürich – including Silvia Brochet as first author – report a new model for studying how dietary resources regulate microbial communities in the gut of honey bees (Brochet et al., 2021).

The Western honey bee, *Apis mellifera*, has several advantages as a model system for studying the coexistence of related microbes. First, its diet consists of pollen and nectar (a mix of simple sugars, complex carbohydrates and proteins) which can be easily replicated in the laboratory. Second, 95% of the bacterial species in their gut belong to the same family which is called *Lactobacillus* Firm-5 (Kwong and Moran, 2016). Most of these microbial communities contain several genetically distinct Firm-5 species which reside in the bee’s rectum, where they consume a diet of pollen (Ellegaard and Engel, 2019).

To find out how closely related bacteria are maintained in the gut, Brochet et al. created an artificial microbial community that contains four Firm-5 species that are commonly found in all Western honey bees. These species were then grown in the guts of live honey bees that had been depleted of their gut microbiome or cultured in the laboratory. The experiments showed that the four species coexisted when the honey bees were fed a diet of pollen but not simple sugar, and this effect was also observed in vitro. On a diet of simple sugar, one species outcompeted all the others; the other three species reached higher population densities when they were grown on their own on pollen or sugars instead of in a community. These results suggest that competition for resources shapes how the different species behave in the community, but these distinct behaviors allow the bacteria to coexist when grown on pollen.

To reveal the mechanisms underlying this coexistence, Brochet et al. combined transcriptomics and metabolomics to investigate how each species consumed the different nutrients derived from pollen. Surprisingly, despite all four species having a similar genetic make-up, they rarely activated the same genes, suggesting that transcriptional regulation may constrain the bacteria from using the resources in pollen in the same way. This was supported by metabolomics which showed that each species consumed a different composition of metabolites: about a third of measured metabolites were consumed by only one species, while a third were consumed by more than one, and the last third were consumed by all four. Furthermore, bacterial species that used the same resources often consumed these at different rates.

Together, these experiments demonstrate that species in the Firm-5 community consume distinct but overlapping profiles of nutrients derived from the pollen diet of bees (Figure 1). This supports the idea that resource partitioning allows closely related microbes to coexist in the gut. A more unexpected observation is the extent to which species can overlap with respect to their functional gene content and still live together. This result highlights the importance of incorporating knowledge of transcriptional regulation and cellular physiology when studying the interactions of closely related bacteria.

**Figure 1.**
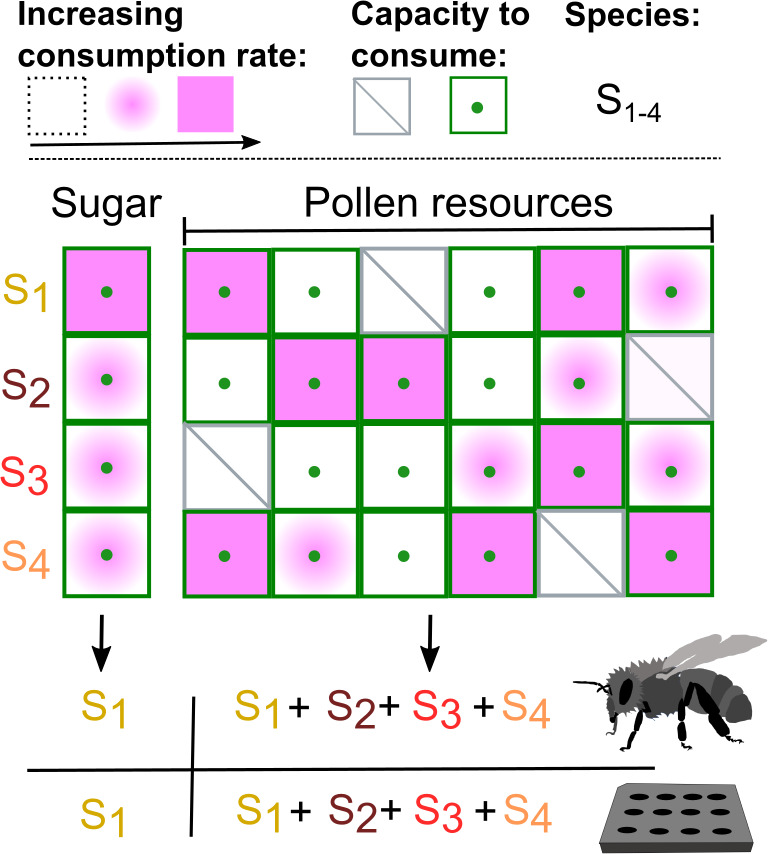
How a diet of pollen allows closely related species of bacteria to coexist in the gut of honey bees. Brochet et al. created an artificial microbial community made up of four species that are commonly found in the gut of honey bees (S_1_, S_2_, S_3_, S_4_) and studied the growth of this model community in vivo (bee symbol) and in vitro. When grown with just one resource (sugar) available, one species (S_1_ in this instance) consumed the resource at a faster rate than the other species (see key at top left), even though all four species were capable of consuming sugar (as indicated by green dots). However, pollen offers multiple nutrients (as represented by the six columns in the figure), which the four species of bacteria consume in different ways. For example, S_3_ is unable to consume the nutrient represented by column 1 (indicated by a diagonal line), but can consume the nutrients represented by the other five columns: moreover, it consumes some nutrients at a higher rate than other species. If the consumption profiles of the four species complement each other (as is the case for the four species studied), they can coexist when grown in the gut of honey bees fed a diet of pollen or when cultured on pollen in the laboratory.

The study by Brochet et al. opens several exciting avenues for future study. The system could be used to measure the degree of diversity required for multiple species to live in a single community: studies that systematically increase species-level diversity are likely to provide a sense of the ‘upper bound’ for this system. In addition, the in vivo and in vitro bee gut models provide an opportunity to examine how microbes with overlapping resource preferences behave in a community.

Further characterization of other Firm-5 species is needed to address which resource preferences and consumption behaviors are more ecologically stable than others. In addition, it would be interesting to compare resource partitioning in the bee gut to culture-based models of competition among closely related bacteria, such as *Bacteroidetes* in the human gut microbiota (Tuncil et al., 2017); this may reveal generic strategies of resource partitioning in gut microbial communities. Importantly, the establishment of the Firm-5 model presents an exciting opportunity for ‘cross-pollination’ between theory and experiment to understand the link between consumption and composition in gut microbial communities.
